# Long-term results of functional endoscopic sinus surgery in children with chronic rhinosinusitis with nasal polyps

**DOI:** 10.1186/2045-7022-3-S2-P34

**Published:** 2013-07-16

**Authors:** Marjolein Cornet, Christos Georgalas, Susanne Reinartz, Wytske Fokkens

**Affiliations:** 1 AMC, Amsterdam, the Netherlands; 2 AMC, ENT, Amsterdam, the Netherlands

## Background

Chronic rhinosinusitis with nasal polyps (CRSwNP) is rare in children and has a major impact on Quality of Life (QoL). Functional endoscopic sinus surgery (FESS) has proven to be an effective treatment, but it is still unclear what long term outcomes are in children with CRSwNP. Therefore the objective of this study was to assess long term results of FESS in children with CRSwNP.

## Methods

In this combined prospective and retrospective study a QoL questionnaire was send to all children who received FESS because of CRSwNP between the year 2000 and 2010. Almost half of these children also filled in this questionnaire preoperatively. Primary outcome was R-SOM score.

## Results

44 Children underwent FESS. From 18 patients we also prospectively collected preoperative QoL questionnaires. The response rate was 82% (36/44) and mean follow-up period was 4.0 years (**±** 2.9). The mean age at surgery was 13 years (**±**2.9). Of these children 9 had CF (25%) and 10 children asthma (28%). R-SOM scores showed a significant improvement both in general symptoms as well as several different domains when comparing pre- and postoperative questionnaires (p=0.04). Only 14% (5) of the patients needed a subsequent intervention. In children with CF this was 33% (3/9).

## Conclusions

This study demonstrates that long term results of FESS in children with CRSwNP are good. Overall QoL has improved significantly for the whole group, especially in nasal symptoms, showing that FESS is a good treatment in children with CRSwNP. Furthermore even children with CF show good results.

